# A Comparative Histomorphometric Analysis of Two Biomaterials for Maxillary Sinus Augmentation: A Randomized Clinical, Crossover, and Split-Mouth Study

**DOI:** 10.1155/2022/4577148

**Published:** 2022-06-02

**Authors:** Carlos Ricardo de Queiroz Martiniano, Lídia Audrey Rocha Valadas, Jose Ronildo Lins do Carmo Filho, Ana Paula Negreiros Nunes Alves, Mara Assef Leitão Lotif, Bruno Salles Sotto-Maior, Thereza Cristina Farias Botelho Dantas, Luciane Lacerda Franco Rocha Rodrigues, Carlos Eduardo Francischone

**Affiliations:** ^1^São Leopoldo Mandic Institute and Research Center, São Paulo, Brazil; ^2^Dentistry and Nursing School, Federal University of Ceara, Fortaleza, Ceara, Brazil; ^3^Ceara Academy of Dentistry, Fortaleza, Ceara, Brazil; ^4^Department of Community and Preventive Dentistry, University of Buenos Aires, Buenos Aires, Argentina; ^5^Department of Restorative Dentistry, Federal University of Juiz de Fora, Juiz de Fora, Minas Gerais, Brazil; ^6^Paulo Picanço College of Dentistry, Fortaleza, Brazil; ^7^Unichristus, Fortaleza, Brazil

## Abstract

**Introduction:**

Considering oral rehabilitation with dental implants, many studies have aimed at improving bone regeneration through the use of biomaterials.

**Objective:**

This study aimed at comparing bone neoformation in patients undergoing bilateral maxillary sinus surgery with two bovine biomaterials.

**Materials and Methods:**

This is a randomized, blinded, clinical crossover, and divided mouth study. Ten participants with an indication of maxillary sinus enlargement were selected and underwent surgical treatment with Bio-Oss® graft biomaterial (graft 1) on one side and Lumina-Porous® graft biomaterial (graft 2) on the other. The samples were collected after nine months and fixed and then decalcified in 10% ethylenediamine tetra-acetic acid (EDTA) solution for 30 days to process and make histological slides. Connective and bone tissue were further analyzed to identify the amount of newly formed bone.

**Results:**

The graft 1 group had a greater formation of vital mineralized tissue when compared to the graft 2 group (*p* = 0.01). For nonvital mineralized tissue and amount of connective tissue, there was no statistical difference (*p* = 0.21 and *p* = 0.09, respectively). The medullary spaces were larger in the graft 2 group. The group treated with graft 1 presented a higher percentage of osteoclasts and viable osteocytes compared to the graft 2 group (*p* = 0.014 and *p* = 0.027, respectively).

**Conclusion:**

Every day, new alternative biomaterials are offered as an option in oral rehabilitation. In this study, both treatments induced bone neoformation after 9 months; however, the group treated with Bio-Oss® showed a higher percentage of vital mineralized bone tissue.

## 1. Introduction

Technical-scientific advancements in dentistry have enabled an evolution by means of oral rehabilitation with the use of osseointegrated implants as an alternative to removable total and partial dentures [1, 2].

The interest of researchers in the search for new natural or synthetic substances which could replace lost bone tissues significantly increased after presenting scientific evidence of osseointegration in the late 1970s which made implantology viable [1]. However, one of the factors for rehabilitation success with dental implants is the quantity and quality of bone tissue present, favoring (or not) the treatment. In situations where bone conditions are not favorable, the dental surgeon can use bone reconstruction techniques to increase the volume and bone quality of the prosthetic space for subsequent installation of the implants [2–4].

Autogenous bone has been the most used material in preprosthetic rehabilitation surgery and treatment of bone defects for restoring function and aesthetics in dentistry. New biomaterials have emerged as research has progressed over the years [4–6]. One of the main definitions for biomaterials is “any and all material, natural or synthetic, which acts on tissues/organs with the objective of replacing a lost bone defect and its function” [7]. In implantology, biomaterials can be considered autogenous when the graft is harvested from the individual to be treated; allogenous/homogeneous when taken from another individual of the same species; and xenogenous/heterogeneous when taken from other species [8].

The autogenous bone graft is still considered the gold standard, as it presents better results for jaw rehabilitation due to its osteogenic, osteoconductive, and osteoinductive properties [9, 10]. However, surgeries with autogenous bone grafts require the need for a second surgical site, the donor site, with the bone normally harvested from the mandibular branch, chin, skullcap, iliac crest, or tibia. In addition to making the surgical process more complex, the need for a second surgical site can lead to sequelae for the patient [10].

As an alternative to autologous bone grafting, the use of xenogeneic graft materials is increasing. This is due to the decrease in morbidity and the reported efficacy and safety of these materials [6]. Xenogeneic grafts do not have living cells since they undergo purification processes, but they can present osteoconductive or osteoinductive characteristics. The great advantage of using these two types of graft is that the second surgical site, the donor site, is dispensed with when working with an autogenous graft, which makes the reconstructive procedure faster, safer, and less complex [5].

In this context, the biomaterial for bone grafting should ideally be osteogenic, osteoinductive, and osteoconductive; be biologically inert; and have rapid revascularizing activity [5, 8]. Among bone grafts in dentistry, xenogenous grafts of bovine origin predominate on the world market due to their biocompatibility and osteoconductive properties [11].

Bio-Oss^®^ biomaterial (Geistlich Pharma AG, Wolhusen, Switzerland) stands out in this area, being widely used in maxillary sinus lifting surgeries, and eventually considered a reference standard as a xenogenous reconstructive material [12]. It is a deproteinized bovine bone mineral with osteoconductive properties which acts as a matrix for bone neoformation. Its effectiveness has been the topic of several studies in the periodontics and implantology areas [9]. It is obtained from the mineral phase of bovine bone. The organic phase is harvested during the manufacturing process, resulting in an intercrystalline structure of microtubules and microcapillaries between the apatite crystals. The resulting matrix resembles human bone in terms of composition, morphology, and ultrastructure [13]. Bio-Oss^®^ has a structure with high porosity which increases its internal surface area and a calcium-phosphate index of 2.03 which combines with human bone [9]. It stimulates bone cell migration through a pore system and encourages osteoblastic differentiation [8]. Bone neoformation occurs with the presence of osteocytes and angiogenesis after grafting with Bio-Oss^®^ in the maxillary sinus. This biomaterial has good biocompatibility without promoting a foreign body reaction. Its excellent osteoconductive properties have high documented implant survival rates, thereby making its use for maxillary sinus lifting safe and presenting predictable results [13].

Another biomaterial, Lumina-Porous^®^ (Criteria Ind. e Com. Ltda., São Carlos, Brazil), is a deproteinized bovine bone mineral produced in Brazil. This is also a biomaterial with high porosity and osteoconductive properties, which helps with bone neoformation and angiogenesis [13]. The sterilization process of this biomaterial occurs by irradiation with gamma rays (25 kGy), and the manufacturing process stabilizes the crystallographic profile of hydroxyapatite. Its biocompatibility is associated with its physiological pH (pH = 6) and its chemical composition of 58% CaO, 40% P_2_O_5_, 1% MgO, and 1% Na_2_O [13]. Lumina-Porous^®^ contains macropores—size between 70 *µ*m and 240 *µ*m—typical features of Haversian canals, and small internal medullary vascular channels of bone. This characteristic results from a porosity percentage between 79% and 85% per granule, something that favors the absorption of endogenous proteins, growth factors, and considerable stabilization of the clot due to the high access of the internal surfaces of the granules. The use of Lumina-Porous^®^ for lifting the maxillary sinus is effective even in cases where there is little bone remaining, favoring further rehabilitation with dental implants [14].

Since Bio-Oss^®^ has a higher number of studies in the literature which prove its effectiveness in lifting the maxillary sinus and only one clinical study about the alternative material Lumina-Porous^®^, the present study aims at comparing the bone neoformation in patients undergoing bilateral maxillary sinus surgery with two bovine biomaterials, thus seeking greater evidence for an alternative material which is more economically accessible.

## 2. Materials and Methods

### 2.1. Study, Location, and Ethical Aspects

This study is a clinical, blind, randomized crossover, and split-mouth study. This research was approved by the research ethics committee of São Leopoldo Mandic School of Dentistry under number 2,019,997. The clinical stage was performed at the Implantology Clinic of the Ceará Academy of Dentistry and the laboratory stage at the Oral Pathology Laboratory of the Federal University of Ceará.

### 2.2. Sample and Randomization

Ten participants who sought treatment with dental implants and written informed consent were recruited to perform the surgeries. They had a mean age of 57.4 ± 8.3 years, with 6 being females and 4 being males. The sample size has been calculated using G*∗*Power 3.1.5 considering a repeated-measures analysis of variance (ANOVA) and a significance level of 5%, and a total of eight individuals would be necessary to achieve a power of 90%.

The inclusion and exclusion criteria of the study are presented in Table 1.

Each participant selected for the study went through a detailed anamnesis, seeking to assess their medical history, smoking habits, and oral cavity examination. Tomographic, biochemical, and hematological exams were requested from all participants.

Randomization was performed in the Excel program to indicate the side of the mouth to receive each material in the surgical stage. After randomization, patients underwent surgical treatment with Bio-Oss® graft biomaterial (graft 1) on one side and Lumina-Porous® graft biomaterial (graft 2) on the other.

### 2.3. Surgical Stage

All the surgical procedures were performed by the same operator. One hour before surgery procedures, patients received 2 g of amoxicillin. Participants underwent surgery with a local infiltrative anesthesia, mepivacaine 3%, extraoral antisepsis with 2% chlorhexidine, and mouthwashes with 0.12% chlorhexidine for one minute and apposition of surgical drapes. A linear type incision of 2.5 cm with mucous-periosteal detachment was performed in the alveolar ridge crest region complemented by two relaxing incisions (1.5 cm each), one in the region of the maxillary first molar and another in the region of the maxillary second molar, to expose the anterior wall of the maxillary sinus.

Osteotomy to delimit the bone access window to the sinus was done with 6 diamond spherical drills mounted on an implant motor (900 rpm) under irrigation with 0.9% physiological saline solution from 2 to 3 mm vertically above the bone crest. Curettes were used to lift the sinus to displace the membrane from the maxillary sinus walls, thus creating an adequate space to accommodate the graft 1 biomaterials on one side and graft 2 on the other side (0.5 g each side). Each sample of the material used had a size corresponding to the weight of 0.5 g. A collagen membrane (Criteria^®^) was then installed after filling the entire space, the muco-periosteal flap was repositioned, and the suture was interrupted without simple stitches with 4–0 silk thread. Patients received antiinflammatory medication (nimesulide 100 mg every 12 hours for 3 days), analgesic (paracetamol 750 mg/8 hours/5 days), and antibiotics (amoxicillin 500 mg/8 hours/7 days).

The patients were followed up, and computed tomography was performed after 9 months to assess the height and thickness available for installing the implants in the grafted area. Patients received the same antiinflammatory medication, analgesic, and antibiotics. Postoperative instructions included liquid/soft diet and the use of 0.12% chlorhexidine mouthwash for 15 days until the sutures were removed. The tissue samples were harvested vertically for histological and histomorphometry analysis nine months after grafting using a 4 mm trephine drill, and samples were collected at the second intervention in the region of the second premolar and first molar at the moment of dental implant installation.

### 2.4. Histopathological and Histomorphometry Analysis

After a period of fixation in buffered 10% formaldehyde (24 to 48 hours), the samples were decalcified (suspended) in 10% ethylenediamine tetraacetic acid (EDTA) solution (pH 7.3) for 30 days to process and make histological slides. Dewaxing was performed, and then 3 *µ*m sections were rehydrated for hematoxylin-eosin staining. Connective and bone tissues were further analyzed to identify the amount of newly formed bone. The number of empty spaces for osteocytes and viable cells was also counted since osteocytes are the main cells associated with maintaining bone vitality. The slide images were digitized using LAS software. 4.1 (Leica Microsystem Image Solutions, Wetzlar, Germany). ImageJ software was used for histomorphometry analysis. The different tissues were isolated by the program, and histomorphometry was performed, accounting for the percentage corresponding to each area. Three types of samples were separated: the newly formed bone tissue, the connective tissue, and the biomaterial residue. The number of vital osteoclasts and osteocytes was also measured. The sum of the fields of each slide was considered as a sample unit, and the percentage of gaps in the empty osteocytes was used for quantitative evaluation.

### 2.5. Statistical Analysis

The collected data were subjected to statistical tests. Intergroup comparisons were performed using Student's *t*-test and analysis of variance. A significance level of 5% was adopted.

## 3. Results

A total of ten patients with indication for maxillary sinus lift (4.23 mm ± 0.62) × (3.00 mm ± 0.89) received treatment with one of the biomaterials on each side. After 9 months, there was no statistical difference between the height and bone width of different participants on histopathological analysis (*p* > 0.05).

**Figure 1:**
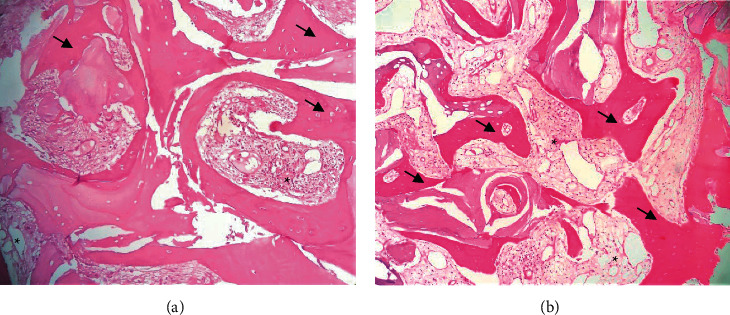
Histopathological and histomorphometric analysis of the tissues corresponding to the two grafts used (H and E). (a) Graft 1 (biomaterial group): regular-shaped trabeculae of woven bone (arrow) in a scarce fibrous stroma (asterisk). (b) Graft 2 (biomaterial group): fine trabeculae of bone tissue (arrow) within paucicellular fibrous connective tissue (asterisk) (hematoxylin-eosin staining 100x).

**Figure 2:**
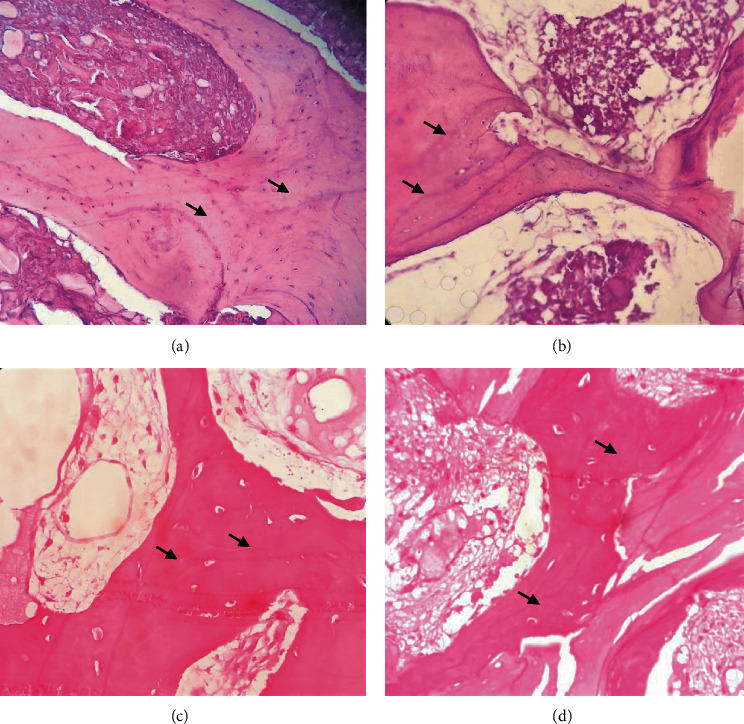
New bone formation comparing the two grafts (histological analysis—H and E). 40x magnification of the histopathological graft 1 biomaterial (a, c) and graft 2 (b, d). *Note.* The greater amount of neoformed bone tissue (arrow) with the use of graft 1 when compared to graft 2 (hematoxylin-eosin staining).

The histopathological analysis of both biomaterials showed the presence of connective tissue, mineralized tissue, and cells such as osteoblasts and osteocytes. These findings can be seen in Figures 1 and 2. The graft 1 group demonstrated the formation of fibrous connective tissue with a better organized structural framework and neoformed bone tissue. Furthermore, there is a higher percentage of viable osteoclasts and osteocytes than in the graft 2 group. Finally, in the graft 1 group, we could observe less spinal cord spaces.

### 3.1. New Bone Formation and Connective Tissue

**Figure 3:**
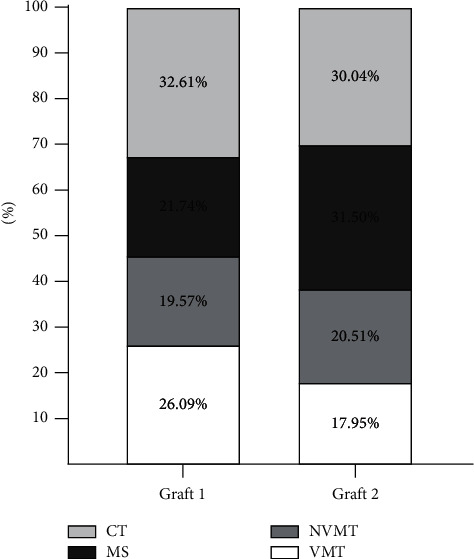
40x magnification of the histopathological graft 1 biomaterial on the left and graft 2 on the right. *Note.* The greater amount of neoformed bone tissue (arrow) with the use of graft 1 when compared to graft 2 (hematoxylin-eosin staining). Legend: percentage of vital mineralized tissue (VMT) and nonvital mineralized tissues (NVMTs), medullary spaces (MS), and connective tissue in graft 1 (left) and graft 2 (right) biomaterials.

In Figure 3, the side treated with graft 1 can be seen to have a greater formation of vital mineralized tissue when compared to the results of graft 2 (*p* = 0.01). The data for the nonvital mineralized tissue were similar, without statistical difference (*p* = 0.21), and the amount of connective tissue was also not significant (*p* = 0.09). The medullary spaces were larger in graft 2 without a statistical difference (*p* = 0.09). The amount of mineralized tissue and connective tissue was calculated using the method of selecting the area of the analyzed tissue, and this area was converted into a percentage value, using ImageJ software.

### 3.2. Osteocyte and Osteoclast Content

**Figure 4:**
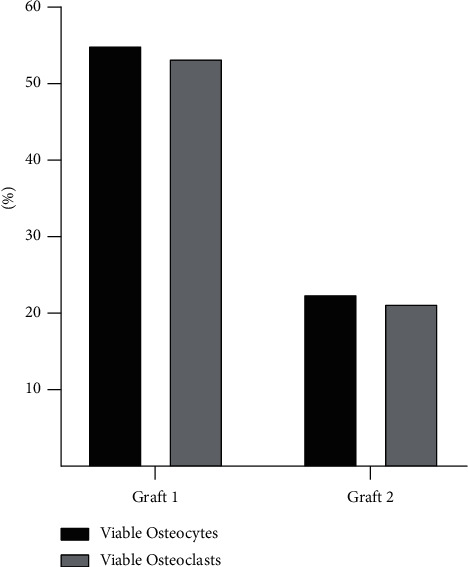
Percentage of the presence of viable osteoclasts and osteocytes in the graft 1 (left) and graft 2 (right) biomaterials.

Figure 4 shows the number of viable osteoclasts and osteocytes found in the histomorphometry analysis of the studied biomaterials. A higher percentage was observed in the group treated with graft 1, with a statistical difference regarding the amount of osteoclasts (*p* = 0.014) and viable osteocytes (*p* = 0.027) found in the graft 2 group. These cell groups were individually quantified by counting using ImageJ software.

## 4. Discussion

The present study compared two similar bovine biomaterials of different countries in lifting the maxillary sinus by means of histomorphometry analysis in a split-mouth design. Nine months after the surgery, it was observed that the maxillary sinus treated with graft 1 presented a great amount of vital mineralized tissue besides osteoclasts and viable osteocytes significantly.

The new bone formation promoted by graft 1 presents strong scientific evidence. Several studies since 1999 have compared graft 1 with other biomaterials through histopathological and histomorphometry analyses in guinea pigs and humans. The results on its biocompatibility and osteoconduction have enabled predictability in results and safety in the use of this biomaterial [8, 15–17].

In a split-mouth study, Mummolo et al. [17] compared graft 1 to Laddec (BioHorizons), another biomaterial derived from bovine bone, in a maxillary sinus survey. Both materials promoted bone neoformation, with a mineralized tissue percentage around 40%, thus constituting similar results to those found in the present study considering the percentages obtained from vital mineralized tissue and nonvital mineralized tissue. Reis et al. [11] used graft 1 and Osseus^®^ (SIN), also derived from bovine bone, for lifting the maxillary sinus and subsequent rehabilitation with implants. The results showed that both materials were integrated into the sinus area, with the evidence of osteoconduction by histopathological evaluation. After one year of follow-up, the patients clinically showed healthy gums and aesthetic and functional maintenance of prosthetic rehabilitation. Cordaro et al. [18] compared graft 1 to Straumann^®^ Bone Ceramic, a biphasic calcium-phosphate biomaterial, in maxillary sinus lifting surgeries, observing similar efficacy and amount of newly formed bone for both biomaterials. Graft 1 has also shown good results when compared to autogenous bone for lifting the maxillary sinus, as in the study by Jo et al. [19] which did not present a significant difference between the groups.

It is noticed that several studies have evaluated the properties of graft 1, both by clinical and histological parameters; however, few research works have studied graft 2.

Goulart and Moraes [13] compared the performance of graft 1 to that of graft 2 in maxillary sinus lift surgery in a split-mouth design with subsequent insertion of implants. They observed that both treatments provided bone conditions which enabled inserting the implants. However, the study only evaluated two patients, without a long-term follow-up. The authors reinforce that the use of graft 1 is well established in the literature, and further studies which indicate scientific evidence for graft 2 are important.

In another study, Rangel Goulart et al. [14] reported a clinical case of maxillary sinus elevation with graft 2 with subsequent implant insertion. The authors argue that the use of the graft 2 biomaterial in the maxillary sinus, even in cases where there is little bone left, can produce successful results which enable osseointegration of dental implants. It can be noted as evidence that both biomaterials evaluated promoted bone neoformation in the present study; however, the formation of vital mineralized tissue was more evident in graft 1. In addition, the percentage of viable osteoclasts and osteocytes was also evaluated, with positive statistical significance in relation to graft 1.

Da Silva et al. [12] compared the performance of graft 1 to that of graft 2 through clinical, radiographic, and histomorphometry data of 13 split-mouth patients who underwent maxillary sinus surgery and rehabilitation with implants. Both biomaterials promoted new bone formation with no difference between the total bone volume and connective tissue between the groups. The implant survival rate in this study was 100% for graft 1 and 88% for graft 2 after three years of follow-up.

On our study, both materials promoted bone neoformation without adverse reactions. However, graft 1 showed better results in relation to bone neoformation. One hypothesis for the findings is that the size of the particles influenced the biomaterial performance since it is more conducive to greater bone neoformation with the use of grafts with particular widths [17].

It is known that the quality of the xenogenous graft of bovine origin depends on its manufacturing process, which makes it mandatory that new products be evaluated for their physical-chemical and biological properties, in addition to clinical effectiveness [11]. The properties of graft 1 and its long-term safety and clinical efficacy are strongly elucidated in the literature. However, as previously mentioned, this cannot be seen for graft 2, as it is a relatively new brand.

The present study has the limitations of not having an implant insertion and prosthetic rehabilitation phase and the short follow-up time. In agreement with Silva et al.'s study, more comparative studies implementing a randomized split-mouth clinical trial are needed since although they present the same raw material, different products may present different behaviors in the long-term and after prosthetic rehabilitation.

In addition, it is extremely relevant that a product on the market is well supported by scientific literature that proves its long-term effectiveness and characteristics as well as comparison with other biomaterials besides the use in maxillary sinus lift surgeries.

## 5. Conclusion

Every day, new alternative biomaterials are offered as an option in oral rehabilitation. In this study, both treatments induced bone neoformation after 9 months; however, the group treated with Bio-Oss® showed a higher percentage of vital mineralized bone tissue.

## Figures and Tables

**Table 1:** Inclusion and exclusion criteria from the study.

*Inclusion Criteria:*
(i) Individuals in need of bilateral maxillary sinus lift (up to 5 mm in height of the maxillary crest were considered)
(ii) Both genders without age restrictions
(iii) Norm systemic
(iv) Good oral health, without dental caries or periodontal disease
(v) Alveolar bone crest height of a maximum of 5 mm bilateral.

*Exclusion criteria:*
(i) Smoking
(ii) Alcoholism
(iii) Use of illicit drugs
(iv) History of allergies
(v) Presence of sinus disorders
(vi) Individuals who have undergone chemotherapy or radiation therapy
(vii) Individuals who have used bisphosphonate

## Data Availability

The data used to support the findings of this study are included within the article.
